# Rice-Fish Farming Improved Antioxidant Defences, Glucose Metabolism, and Muscle Nutrient of *Carassius auratus* in Sichuan Province

**DOI:** 10.3390/metabo14120710

**Published:** 2024-12-17

**Authors:** Tao Yan, Yun-Yi Xie, Bo Zhou, Xu Kuang, Qing-Zhi Li, Feng-Qi Zhao, Qian-Dong Li, Bin He

**Affiliations:** 1 Fisheries Research Institute, Sichuan Academy of Agricultural Sciences, Chengdu 611730, China; yantao@scsaas.cn (T.Y.); caroline@scsaas.cn (Y.-Y.X.); zhoubo@scsaas.cn (B.Z.); kuangxu@scsaas.cn (X.K.); liqingzhi@scsaas.cn (Q.-Z.L.); zhaofengqi1@scsaas.cn (F.-Q.Z.); liqiandong1@scsaas.cn (Q.-D.L.); 2 Fish Resources and Environment in the Upper Reaches of the Yangtze River Observation and Research Station of Sichuan Province, Chengdu 611731, China

**Keywords:** rice-fish farming, *Carassius auratus*, antioxidant defenses, metabolism, nutrient

## Abstract

Rice-fish farming is an ancient and enduring aquaculture model in China. This study aimed to assess the variations in digestive enzymes, antioxidant properties, glucose metabolism, and nutritional content between *Carassius auratus* reared in paddy fields and ponds. Notably, the levels of amylase and trypsin in *C. auratus* from rice paddies were considerably higher compared to those from ponds. Additionally, the hepatic catalase (CAT) activity in fish from paddy (2.45 ± 0.16 U/mg) exceeded that of their pond counterparts (2.27 ± 0.25 U/mg). Regarding glucose metabolism, the activities of key enzymes such as Na+/K+-ATPase (NKA) (paddy: 82.45 ± 6.11 U/g; pond: 78.53 ± 7.18 U/g), hexokinase (HK) (paddy: 9.55 ± 0.58 U/g; pond: 8.83 ± 0.72 U/g), glucokinase (GK) (paddy: 4.09 ± 0.21 IU/g; pond: 3.44 ± 0.33 IU/g), glucose-6-phosphatase (G6Pase) (paddy: 85.71 ± 4.49 IU/g; pond: 79.12 ± 9.34 IU/g), and glucose-6-phosphate dehydrogenase (G6PDH) (paddy: 47.23 ± 3.22 U/g; pond: 42.31 ± 4.93 U/g) were significantly elevated in rice paddy-cultured fish compared to those in ponds. Conversely, phosphor-pyruvate kinase (PK) (paddy: 418.15 ± 31.89 U/g; pond: 570.16 ± 56.06 U/g) activity was markedly reduced in the paddy group. Hepatic glycogen content (paddy: 15.70 ± 0.98 ng/g; pond: 14.91 ± 1.24 ng/g) was also substantially higher in fish from paddy, although no significant differences in muscle glycogen content (paddy: 7.14 ± 0.59 ng/g; pond: 6.70 ± 0.52 ng/g) were observed between the two environments. In terms of nutritional composition, fish raised in paddy exhibited higher crude protein (paddy: 18.46 ± 0.47 g/100 g muscle; pond: 15.57 ± 0.25 g/100 g muscle) and crude ash (paddy: 1.19 ± 0.02 g/100 g muscle; pond: 0.97 ± 0.02 g/100 g muscle) than those in ponds, whereas the crude fat (paddy: 0.87 ± 0.04 g/100 g muscle; pond: 1.66 ± 0.04 g/100 g muscle) was notably lower in paddy fish. Furthermore, fish from rice paddies had a greater total content of monounsaturated fatty acids (MUFA) (paddy: 4.25 ± 0.24 g/100 g muscle; pond: 6.73 ± 0.27 g/100 g muscle), non-essential amino acids (NEAA) (paddy: 9.04 ± 0.3 g/100 g muscle; pond: 7.19 ± 0.21 g/100 g muscle), and delicious amino acids (DAA) (paddy: 7.11 ± 0.2 g/100 g muscle; pond: 5.45 ± 0.19 g/100 g muscle) compared to those from pond cultures. These findings suggest that rice-fish co-culture systems can yield healthier and more environmentally sustainable aquatic products by improving feed digestion and optimizing nutrient metabolism.

## 1. Introduction

Aquaculture is the practice of cultivating aquatic animals and plants in controlled or semi-controlled environments, which is the important source of high-quality protein products and plays a crucial role in maintaining the global food supply [1]. However, intensive aquaculture has been shown to have some problems, and further optimization of the aquaculture model is necessary, such as wastewater [2].

Traditional agricultural systems were developed, shaped, and maintained by generations of farmers who adopted management practices tailored to local conditions [3]. Reviewing the ecological legacy of traditional farming systems and integrating these experiences into the design of future farms can help to develop a more sustainable agriculture model. The rice-fish farming system, an ancient and sustainable aquaculture model, has been a staple in Chinese agricultural practices for over 1200 years [4]. This integrated approach to agriculture not only optimizes land and water resource utilization but also enhances biodiversity and ecological balance [5,6]. In recognition of its ecological and cultural significance, the Chinese rice-fish coculture system was designated as a Globally Important Agricultural Heritage System (GIAHS) in 2002 by the Food and Agriculture Organization (FAO), the United Nations Development Program (UNDP), and the Global Environment Facility (GEF) [7]. This designation underscores the system’s role as a model for sustainable agriculture that harmonizes food production with environmental conservation.

*Carassius auratus*, a member of the *Cyprinidae* family, is widespread throughout the Eurasian continent. With the advantages of good taste, fast growth, and suitability for mono- and poly-culture, *C. auratus* has been one of the most intensively cultured freshwater fish in China [8]. According to the China Fishery Statistical Yearbook [9], *C. auratus* had already contributed to 2.74 million tons in 2020. However, traditional pond aquaculture has several issues, including poor habitat, land resource occupation, and environmental pollution, and it has become a consensus to explore a new aquaculture model [10,11,12]. Practice has shown that a variety of economic aquatic animals were suitable for the rice-farming system, such as fish, crab, crayfish, and turtles [13]. *C. auratus*, with its omnivorous diet and low oxygen tolerance, is well-suited for rice-fish farming [8,13].

Traditional agricultural systems reflect a successful adaptation to different environments and are rich in biological diversity [3]. Rice-fish farming is thought to improve the living environment for fish; for example, rice relieves high temperatures in water during the summer and reduces ammonia-nitrogen levels; thus, fish in rice paddies are more energetic and have more swimming and feeding behaviors [6]. Similarly, many studies have explored the enhancement of aquatic product quality by the rice-fish farming system in the pathways of gut homeostasis, immunity, and stress. Zhou et al. [14] reported that the shrimp muscle composition of the paddy fields presented significantly high protein and low lipid levels. Similarly, Wang et al. [15] reported that rice flowering improved the tilapia muscle nutrients, intestinal microbiota diversity, and disease resistance.

There are few studies on the effects of the rice-fish farming system on the liver antioxidant system, nutrient metabolic system, and muscle nutritional quality of *C. auratus*. Therefore, we compared the differences in liver antioxidant enzyme activities, digestion and metabolism-related enzyme activities, and muscle multiple nutrients between the two culture environments in the hope of providing the theoretical basis to promote the development of rice-fish farming.

## 2. Materials and Methods

### 2.1. Experimental Design

The *C. auratus* bred in 2023 were put into the ponds and paddy fields when the average length was about 10 cm in April. The rice-fish farming system did not allow any commercial diet, and the fish of the paddy fields were fed a commercial diet by TONGWEI Company, Chengdu, China. A total of 78 *C. auratus*, including 20 fish (38.1 ± 11.4 g, mean ± SE) of ponds (PO) and 20 fish (48.5 ± 11.0 g, mean ± SE) of paddy fields (PA), was collected from Nanxi (Yibin, China) in October. All fish handling and experimental procedures were approved by the Animal Care and Use Committee of the Fisheries Research Institute, Sichuan Academy of Agricultural Sciences (Approval No. 20230608001A).

### 2.2. Sample Collection

The fish were anesthetized with 0.1 g/kg MS-222 (Sigma-Aldrich, St. Louis, MO, USA) and executed. Approximately 12 fish from ponds and paddy fields were randomly dissected to obtain tissue samples (intestinal tract, liver, and muscle), and each 4 fish were mixed as a sample. Intestinal tract samples were used to assay amylase, lipase, and trypsin activities. Liver samples were used to assay total antioxidant capacity (T-AOC), malondialdehyde (MDA) content and superoxide dismutase (SOD), glutathione peroxidase (GPx), catalase (CAT), Na+K+–ATPase (NKA), pyruvate kinase (PK), glucokinase (GK), glucose-6-phosphatase (G6pase), and 6-phosphate dehydrogenase (G6PDH) activities. Liver and muscle samples were used to assay glycogen content. Muscle samples were used to assay nutrient composition. All the samples were promptly frozen in liquid nitrogen and stored at −80 °C until analysis.

### 2.3. Enzymatic Analysis

For the purpose of conducting enzymatic assays, the designated samples were meticulously measured and then thoroughly mixed with chilled phosphate-buffered saline (PBS) at a pH level of 7.4, ensuring a sample-to-saline ratio of 1:9. This process was carried out on ice utilizing a manually operated glass homogenizer. Post-homogenization, the samples underwent centrifugation at 4 °C with a relative centrifugal force of 3000× *g* for 15 min. The supernatant, which was obtained after centrifugation, was meticulously collected and immediately placed into a 2 mL Eppendorf tube for further enzymatic analysis. The enzymatic activities of amylase (Catalog No. F3818-A, U/g), lipase (Catalog No. F3817-A, U/g), trypsin (Catalog No. F69735-A, IU/g), T-AOC (Catalog No. S01440–1, ng/g), MDA (Catalog No. F6293-A, μmol/g), SOD (Catalog No. F3822-A, IU/mg), GPx (Catalog No. F69036-A, U/g), CAT (Catalog No. F69001-A, U/mg), glycogen (Catalog No. F69119-A, ng/g), NKA (Catalog No. F6448-A, U/g), PK (Catalog No. F69158-A, U/g), HK (Catalog No. F69158-A, U/g), GK (Catalog No. F69155-A, IU/g), G6Pase (Catalog No. F69163-A, IU/g), and G6PDH (Catalog No. F69160-A, U/g) were quantified using commercial fish ELISA kits provided by Fankewei Bio, Shanghai, China, following the manufacturer’s guidelines [16]. The protein content within the tissue samples was ascertained by employing the bicinchoninic acid (BCA) protein assay reagent, adhering to the protocol described by Bradford [17].

### 2.4. Assessment and Evaluation of Dorsal Muscle Nutrient Composition

The basal nutrient composition of the dorsal muscle was assayed using the standard methods prescribed by the Association Official Analytical Chemists [18]. Moisture was determined by drying at 105 °C until constant weight. Crude protein was assayed by measuring nitrogen using the Kjeldahl method. Crude lipid was analyzed by combustion in a muffle furnace at 550 °C for 12 h. Amino acids were analyzed by the hydrochloric acid hydrolysis method. Fatty acid composition was determined by gas chromatography/mass spectrometry (GC/MS) with reference to the NIST08 standard spectral library and characterized according to the order of peaks. The relative percentage content of each major fatty acid was calculated by the area normalization method.

AAS was used to evaluate the amino acid composition according to the model of amino acid score criteria recommended by the Food and Agriculture Organization of the United Nations/World Health Organization (FAO/WHO) [19]. The ratio of branched-chain amino acids to aromatic amino acids (Fischer ratio) was used to evaluate the quality of amino acids.

(1) $$AAS=\frac{mg\;of\;amino\;acid\;in\;1\;g\;of\;test\;protein}{mg\;of\;amino\;acid\;in\;reference\;pattern}\times 100$$

(2) $$Fischer\;ratio=\frac{Val+Leu+Ile}{Phe+Tyr}$$

Index of atherogenic (IA) and index of thrombogenic (IT) were used to evaluate the nutritional value of fatty acids in dorsal muscle, and polyene index (PI) was used to evaluate the degree of oxidation of polyunsaturated fatty acids.

(3) $$IA=\frac{C12:0+4\times C14:0+C16:0}{\sum MUFA+\sum PUFA}$$

(4) $$IT=\frac{C14:0+C16:0+C18:0}{0.5\times \sum MUFA+0.5\times \sum PUFA\left(n-6\right)+3\times \sum PUFA\left(n-3\right)+\frac{\sum PUFA\left(n-6\right)}{\sum PUFA\left(n-3\right)}}$$

(5) $$PI=\frac{C20:5+C22:6}{C16:0}$$

### 2.5. Data Analysis

Data were presented as means ± standard error (M ± SE). Data were analyzed with multiple T tests by the Holm-Šídák method using GraphPads Prism 9.5.1, and differences between groups were considered significant at *p* < 0.05 [20].

## 3. Results

### 3.1. Activity Levels of Digestive Enzymes

To elucidate the effects of different farming modes on the digestive capacity of *C. auratus*, the activities of three major digestive enzymes, respectively amylase, lipase, and trypsin, were assayed. As the results were shown in Table 1, the activities of amylase (PA: 18.31 ± 1.82 U/g; PO: 17.71 ± 1.23 U/g) and trypsin (PA: 9.79 ± 0.73 IU/g; PO: 9.20 ± 0.58 IU/g) were significantly higher in PA compared with PO (*p* < 0.05). However, the lipase activity did not differ between PA (4.34 ± 0.34 U/g) and PO (4.06 ± 0.25 U/g) (*p* > 0.05).

### 3.2. Activity Levels of Antioxidant Indices

In *C. auratus* liver, the enzymatic activity of CAT was significantly higher in PA (2.45 ± 0.16 U/mg) compared with PO (2.27 ± 0.25 U/mg) (*p* < 0.05). However, the levels of T-AOC (PA: 4.29 ± 0.34 ng/g; PO: 4.18 ± 0.34 ng/g) and MDA (PA: 1.11 ± 0.05 μmol/g; PO: 1.11 ± 0.12 μmol/g) and activity of SOD (PA: 8.91 ± 0.63 IU/mg; PO: 8.55 ± 0.93 IU/mg) and GPx (PA: 58.99 ± 4.63 U/g; PO: 56.42 ± 4.89 U/g) in liver were not significantly different between PA and PO (*p* > 0.05) (Table 2).

### 3.3. Activity Levels of Metabolish Indices

Liver NKA (PA: 82.45 ± 6.11 U/g; PO: 78.53 ± 7.18 U/g), HK (PA: 9.55 ± 0.58 U/g; PO: 8.83 ± 0.72 U/g), GK (PA: 4.09 ± 0.21 IU/g; PO: 3.44 ± 0.33 IU/g), G6Pase (PA: 85.71 ± 4.49 IU/g; PO: 79.12 ± 9.34 IU/g), and G6PDH (PA: 47.23 ± 3.22 U/g; PO: 42.31 ± 4.93 U/g) activities were significantly higher in PA compared to PO (*p* < 0.05), while liver PK activity was significantly lower in PA (418.15 ± 31.89 U/g) than in PO (570.16 ± 56.06 U/g) (*p* < 0.05) (Table 3).

### 3.4. Nutritional Analysis of Dorsal Muscle

To reveal the effects of muscle nutrition of *C. auratus* under the two culture models, the basic nutrient components, amino acids, and fatty acids in the dorsal muscle were focused on. As shown in Table 4, crude protein (PA: 18.46 ± 0.47 g/100 g muscle; PO: 15.57 ± 0.25 g/100 g muscle) and crude ash (PA: 1.19 ± 0.02 g/100 g muscle; PO: 0.97 ± 0.02 g/100 g muscle) were significantly higher in PA compared with PO (*p* < 0.05). On the contrary, crude fat was significantly lower in PA (0.87 ± 0.04 g/100 g muscle) compared with PO (1.66 ± 0.04 g/100 g muscle) (*p* < 0.05). Furthermore, we performed a comparative assessment of hepatic and muscle glycogen content. The data revealed a statistically significant increase in liver glycogen content within the PA group (15.70 ± 0.98 ng/g) compared to the PO group (14.91 ± 1.24 ng/g) (Table 4). In contrast, no significant difference was observed in muscle glycogen content (PA: 7.14 ± 0.59 ng/g; PO: 6.70 ± 0.52 ng/g) (Table 4).

Fatty acid composition and nutritional value evaluations were demonstrated in Table 5. There were 18 fatty acids detected in the PA group, including 5 SFAs, 3 MUFAs, and 10 PUFAs. Additionally, 20 fatty acids were detected in the PA group, including 6 SFAs, 4 MUFAs, and 10 PUFAs. The total amount of MUFA was significantly lower in PA (4.25 ± 0.24 g/100 g muscle) compared with PO (6.73 ± 0.27 g/100 g muscle) (*p* < 0.05). Fatty acid nutritional evaluation showed that the values of IA (PA: 0.42 ± 0.01; PO: 0.26 ± 0.01), IT (PA: 0.47 ± 0.01; PO: 0.34 ± 0.03), and PI (PA: 0.41 ± 0.01; PO: 0.33 ± 0.03) were significantly higher in PA compared with PO (*p* < 0.05).

Amino acid composition was demonstrated in Table 6. The contents of NEAA (PA: 9.04 ± 0.3 g/100 g muscle; PO: 7.19 ± 0.21 g/100 g muscle) and DAA (PA: 7.11 ± 0.2 g/100 g muscle; PO: 5.45 ± 0.19 g/100 g muscle) were significantly higher in PA compared with PO (*p* < 0.05). The nutritional value evaluations of amino acids were demonstrated in Table 7. Valine was the first limiting amino acid of the PA group, while Leucine was the first limiting amino acid of the PO group. In addition, the Fischer ratio was significantly higher in PA (2.09 ± 0.10) compared with PO (1.58 ± 0.02) (*p* < 0.05).

## 4. Discussion

Rice-fish co-culture is an ancient agricultural wisdom [6]. This strategy enhances the utilization of land and water resources, providing humans with grains and meat while reducing the risk of environmental pollution. However, due to the slow development of science in this field, rice-fish co-culture is only implemented in small areas compared to rice monoculture and intensive aquaculture [21,22]. This study focuses on the rice- *C. auratus* co-culture system in Sichuan Province, China, with the expectation of providing a theoretical basis for the green development of the rice-fish farming system.

### 4.1. Rice-Fish Farming Improves Digestive Enzyme Activity in C. Auratus

Fish need to consume a lot of energy during the growth process, and the ability to digest food effectively is the key to its growth performance [23]. The level of digestive enzyme activity directly affects the ability of fish to digest food, and improving the activity of digestive enzymes can effectively improve the utilization of nutrients by fish, which in turn improves their growth performance [24]. The association between rice-fish farming and digestive enzyme activity has been mentioned in some research. Tilapia living in rice paddies have a higher intestinal microbiota diversity, which improves their digestion and absorption, leading to higher growth rates [15]. Crayfish have a more stable intestinal environment in rice paddies, promoting better nutrient absorption [25]. Similarly, another study on crayfish demonstrated that rice-fish symbiosis led to changes in their intestinal microbiota, stimulating the digestive function of the hepatopancreas [14]. In our results, the activities of amylase and trypsin were significantly higher in the PA group compared to the PO group. Compared to ponds, the rice paddy environment was richer in food sources, including insects, weeds, and rice pollen [6,15]. Meanwhile, natural food intake, including plant and animal feeds, is considered more suitable for gut health in omnivorous fish [26]. Although the exact pathway is not yet clear, the higher activities of amylase and trypsin in the intestines of PA group *C. auratus* may be associated with their increased foraging on plants (e.g., weeds and phytoplankton) and insects due to the abundant food present in farmland environments [13,27].

### 4.2. Rice-Fish Farming Influences the Antioxidant Defenses System in C. Auratus

Enzymatic antioxidants, integral components of the body’s antioxidant machinery, include SOD, CAT, and GPx, which play crucial roles in neutralizing excess ROS at various stages [28,29]. ROS, such as H_2_O_2_, OH^−^, and O_2_^−^, are known to induce LPO, thereby disrupting cellular macromolecular functions and enzymatic activities [30]. T-AOC represents the collective antioxidant potential within the organism, with its levels indicating the robustness of the antioxidant defense to a certain extent [31]. SOD, a naturally occurring scavenger of superoxide radicals, catalyzes the conversion of superoxide radicals (O_2_^−^) into H_2_O_2_ [32], whereas CAT facilitates the decomposition of H_2_O_2_ into water (H_2_O) and molecular oxygen (O_2_), thereby effectively eliminating excessive free radicals [33]. GPx safeguards cellular integrity against lipid peroxidation, enhances the breakdown of H_2_O_2_, and detoxifies harmful peroxides into benign hydroxyl compounds [34]. Furthermore, malondialdehyde (MDA), a byproduct of lipid peroxidation, exerts cytotoxic effects. Consequently, MDA levels are frequently employed as a biomarker for assessing the extent of oxidative stress and the efficacy of the body’s antioxidant defenses [35,36].

The liver is an important metabolic and detoxification organ in animals, and oxidative damage to the liver directly affects the health of the organism [37]. In the present research, we found that the activity of hepatic CAT was significantly increased in the PA group. The level of T-AOC and activities of SOD and GPx showed an increasing trend in the PA group, although there was no significant difference. Similar to our results, Chinese mitten crabs (*Eriocheir sinensis*) living in lakes with greater species diversity showed higher CAT activity and lower MDA levels [38]. Changes in the antioxidant system of animals may be caused by a variety of reasons. For instance, Wang et al. [39] reported that changes in diet composition affected total antioxidant capacity and superoxide dismutase activity in Chinese mitten crab. Qiu et al. [40] investigated the effects of water factors on the antioxidant system of largemouth bass (*micropterus salmoides*) and showed that MDA content and lysozyme activity were significantly and positively correlated with nitrite nitrogen content. Compared to aquaculture ponds, paddy has a richer biodiversity, including both animals and plants. The presence of these organisms provides crucian carp with hiding places, a more diverse range of food sources, and a suitable living environment that is more in line with their natural habits, which is beneficial for ensuring animal welfare [27]. This is advantageous for the health of the crucian carp and is reflected in their better antioxidant enzyme activity. The mechanisms by which rice-fish farming affects the antioxidant capacity of fish are still unclear, and further research will help optimize the aquaculture strategies for rice-fish farming systems.

### 4.3. Rice-Fish Farming Influences the Glucose Metabolic Pathway in C. Auratus

In general, teleost fish are unable to rapidly reduce circulating glucose levels after glucose load or a high-carbohydrate diet and are therefore considered glucose-intolerant [41]. However, even taking into account that carbohydrate metabolism in fish appears to play only a secondary role to lipids and proteins [42], carbohydrates remain crucial to the metabolism of all fish. Under aerobic conditions, glucose is metabolized either by the glycolytic pathway, the tricarboxylic acid cycle, and respiratory chain catabolism, which results in the production of ATP, or by the pentose phosphate pathway catabolism, which results in the production of NADPH and ribulose 5-phosphate, required for nucleotide synthesis [43]. Excess glucose may be stored as glycogen. In the fasting state, metabolic glucose requirements may be met by glycogen degradation or by glyconeogenesis for glucose synthesis [44]. Therefore, the present study focused on the glycogen content in the liver and muscle, as well as on the activities of key rate-limiting enzymes involved in the glycolysis (HK, GK, PK), gluconeogenesis (G6Pase), and pentose phosphate pathways (G6PDH).

HK catalyzes the first reaction of glycolysis, phosphorylating glucose to glucose-6-phosphate. The HKs have four members, of which HK-IV, also known as GK, plays a major role in mammalian glucose homeostasis [45]. Increased dietary carbohydrates were observed to improve the activity of the HK family in a variety of fish species, suggesting that the HK family may function as a dietary regulatory isoform [45,46]. For instance, in rainbow trout (*Oncorhynchus mykiss*), gilthead seabream (*Sparus aurata*), and common carp (*Cyprinus carpio*), high dietary carbohydrates increased GK gene expression and activity [45]; in Atlantic salmon (*Salmo salar*), GK gene expression was higher in individuals fed high-carbohydrate diets [46]. In the present study, liver HK and GK activities were increased in the PA group, indicative of enhanced glycolysis activity within the liver. *C. auratus* is an omnivorous fish that can digest and absorb some carbohydrates [47], and the increased activity of glycolytic enzymes may suggest an increase in dietary carbohydrates. There are more plants present in paddy compared to ponds, which provide a greater source of carbohydrates for *C. auratus* [6]. Meanwhile, the enhanced activity of glycolysis also implied that *C. auratus* raised their carbohydrate intake, suggesting that crucian carp may have ingested more plant feeds in the paddy.

PK is another key glycolytic enzyme that catalyzes the last step in glycolysis, the conversion of PEP to pyruvate [48]. In contrast to the trend of the HK family, PK of *C. auratus* in the PA group showed reduced activity. Previous studies have shown that the carbohydrate-fat ratio of feeds affects the activity of glycolytic enzymes and that the type of carbohydrate also affects changes in key enzymes of glycolysis [49]. In common carp, dietary supplementation with 30% starch, glucose, or fructose did not affect liver PK activity compared with fish fed a high-protein diet with no carbohydrates, whereas supplementation with 30% galactose reduced PK activity [50]. Since food sources are more abundant in paddy, changes in PK activity may be related to the type of carbohydrates ingested, suggesting differences in dietary composition between paddy and pond.

G6Pase is a microsomal enzyme that plays a key role in blood glucose homeostasis by catalyzing the dephosphorylation of glucose-6-phosphate to glucose, which is the final step in gluconeogenesis and glycogenolysis [43]. Previous studies have shown that the expression level of G6Pase in the liver is regulated by nutrients. For instance, high-fat or high-protein diets significantly increased G6Pase expression and enzyme activity in rainbow trout [51,52]. Meanwhile, G6Pase was also affected by starvation. In gilthead sea bream (*Sparus aurata*), re-feeding after starvation reduced G6Pase expression, thereby reversing glucose/glucose-6-phosphate substrate cycling flux in the liver [53]. However, the nutritional regulation of G6Pase in fish liver shows significant species differences. Long-term starvation increases G6Pase activity in rainbow trout [54], but decreases it in yellow perch (*Perca flavescens*) [55]. Common carp fed a high-carbohydrate diet lead to suppressed G6Pase activity [50], while the G6Pase in gilthead sea bream is not sensitive to the carbohydrate content in the feed [56]. In the present study, the increased G6Pase activity in the liver of the PA group indicates that the gluconeogenic pathway in the rice paddy *C. auratus* is active. Mei et al. [47] found that in *C. auratus* at 63 days and 340 days of age, the expression of G6Pase in the liver showed an increasing trend with the increase in dietary starch content, but there was no significant difference. Due to the abundance of carbohydrate sources in paddy, *C. auratus* may have increased its intake of carbohydrates, leading to an increase in G6Pase activity in the liver. However, the regulatory mechanisms of the gluconeogenic pathway in *C. auratus* under the rice-fish farming system still require further exploration.

G6PDH is involved in the pentose phosphate pathway of glucose metabolism and is an important rate-limiting enzyme. Through the pentose phosphate pathway, glucose metabolism produces NADPH for the further synthesis of reduced glutathione and thioredoxin, as well as ribose 5-phosphate, which is required for nucleotide synthesis [57,58,59]. G6PDH plays a significant role in lipid synthesis, clearance of ROS, maintaining red blood cell metabolism, and preserving the integrity of cell membranes [60,61]. For example, *Litopenaeus vannamei* infected with parasites show inhibition of G6PDH [62]. Meanwhile, grass carp (*Ctenopharyngodon idella*) that have been consuming Vicia faba (broad beans) over a long period exhibit a decrease in G6PDH expression, leading to hemolysis [63]. Additionally, Deane et al. [64] have reported that up-regulation of the growth axis is associated with increased G6PDH expression in black sea bream adapted to isotonic salinity. In the present study, the G6PDH activity in the PA group was higher than that in the PO group, indicating that the pentose phosphate pathway in the PA is more active. This suggests that *C. auratus* in the PA group is in good health. Similarly, Wang et al. [15] reported that tilapia living in rice paddies grow faster than those in tanks, and this phenomenon may also be associated with the activity of G6PDH.

Overall, the changes in the liver of *C. auratus* indicate that the rice-fish farming system can influence carbohydrate metabolism through glycolysis, gluconeogenesis, and the pentose phosphate pathway. *C. auratus*, being omnivorous, possesses a certain level of glucose tolerance [65]. The rice-fish farming system seems to have developed the potential of *C. auratus* to utilize carbohydrates. The mechanisms behind these changes are not yet clear, but one of the reasons might be the rich food sources in paddy that provide a diet more in line with the natural feeding habits of *C. auratus*.

### 4.4. Rice-Fish Farming Influences the Muscle Nutrient Composition in C. auratus

Muscles are arguably the most important edible part of the fish and the main site of nutrient deposition [66]. The proximate composition of muscle is an important factor in responding to its nutritional value, including protein, lipid, ash, and moisture. The content of these nutrients is influenced by a number of factors, such as environmental conditions, and is reflected in flesh quality. In the present study, we found that crude protein and crude ash content increased while crude fat content decreased in the PA group. Likewise, the findings regarding the impact on proximate composition have been documented in rice-tilapia farming [67]. Rice-fish farming ecosystems have richer biodiversity than traditional aquaculture ponds [67], and rich groups of microbes could serve as additional food for *C. auratus*. In addition, the results of the amino acid profile showed that the content in the dorsal muscles of the PA group was significantly higher than that of the PO group. The muscles of the PA group were rich in delicious amino acids, suggesting that the rice-fish farming model improved the overall flavor of flesh quality. Our results were in accordance with other rice-aquaculture farming systems [25,67,68], in which Inayat suggested that it was related to the feeding of fish in different environments. Fischer ratio is used to assess if the amino acid composition of a food is reasonable, and high F-ratio foods are of interest due to their potential for liver metabolism, anti-fatigue, and antioxidant activity [69]. In the present study, the F-value of the PA group was significantly higher than that of the PO group, suggesting that rice-fish farming could provide aquatic products more favorable to human health. The flavor and tenderness of the flesh were also affected by the content of muscle fatty acids. The present study showed that the MUFA content of the PA group was significantly lower than that of the PO group, which could be related to the full movement of rice *C. auratus*.

## 5. Conclusions

This study compared the effects of rice-fish farming and pond aquaculture on *C. auratus*. The results indicated that rice-fish farming influenced the digestive enzyme activity, antioxidant defense system enzyme activity, liver carbohydrate metabolism, and muscle nutritional components of *C. auratus*. These changes are associated with the rich biodiversity in the rice paddies, which provide a living environment and natural food that are more in line with the natural habits and diet of the *C. auratus*. *C. auratus* from the rice-fish farming system had better meat quality, which is more in line with the needs of a healthy human diet. This study will provide a theoretical basis for promoting the development of rice-fish farming.

## Figures and Tables

**Table 1:** Comparison of digestive enzymes of *C. auratus* cultured in paddy group (PA) and pond group (PO).

	Paddy (PA)	Pond (PO)
Amylase (U/g)	18.31 ± 1.82 ^a^	17.71 ± 1.23 ^b^
Lipase (U/g)	4.34 ± 0.34	4.06 ± 0.25
Trypsin (IU/g)	9.79 ± 0.73 ^a^	9.20 ± 0.58 ^b^

Values in each row with different letters have significant differences (*p* < 0.05), n = 12.

**Table 2:** Comparison of hepatic antioxidant indices of *C. auratus* cultured in paddy group (PA) and pond group (PO).

	Paddy (PA)	Pond (PO)
T-AOC (ng/g)	4.29 ± 0.34	4.18 ± 0.34
MDA (μmol/g)	1.11 ± 0.05	1.11 ± 0.12
SOD (IU/mg)	8.91 ± 0.63	8.55 ± 0.93
GPx (U/g)	58.99 ± 4.63	56.42 ± 4.89
CAT (U/mg)	2.45 ± 0.16 ^a^	2.27 ± 0.25 ^b^

Values in each row with different letters have significant differences (*p* < 0.05), n = 12.

**Table 3:** Comparison of metabolism indices of *C. auratus* cultured in paddy group (PA) and pond group (PO).

	Paddy (PA)	Pond (PO)
NKA (U/g)	82.45 ± 6.11 ^a^	78.53 ± 7.18 ^b^
PK (U/g)	418.15 ± 31.89 ^b^	570.16 ± 56.06 ^a^
HK (U/g)	9.55 ± 0.58 ^a^	8.83 ± 0.72 ^b^
GK (IU/g)	4.09 ± 0.21 ^a^	3.44 ± 0.33 ^b^
G6Pase (IU/g)	85.71 ± 4.49 ^a^	79.12 ± 9.34 ^b^
G6PDH (U/g)	47.23 ± 3.22 ^a^	42.31 ± 4.93 ^b^

Values in each row with different letters have significant differences (*p* < 0.05), n = 12.

**Table 4:** Basic nutrient content of dorsal muscles and liver from *C. auratus* in paddy group (PA) and pond group (PO).

	Paddy (PA)	Pond (PO)
Crude protein (g/100 g muscle)	18.46 ± 0.47 ^b^	15.57 ± 0.25 ^a^
Crude ash (g/100 g muscle)	1.19 ± 0.02 ^b^	0.97 ± 0.02 ^a^
Crude fat (g/100 g muscle)	0.87 ± 0.04 ^a^	1.66 ± 0.04 ^b^
Moisture (g/100 g muscle)	80.45 ± 0.73	80.31 ± 0.58
Liver glycogen (ng/g)	15.70 ± 0.98 ^a^	14.91 ± 1.24 ^b^
Muscle glycogen (ng/g)	7.14 ± 0.59	6.70 ± 0.52

Values in each row with different letters have significant differences (*p* < 0.05), n = 12.

**Table 5:** Fatty acid composition (g/100 g muscle) and nutritional value evaluation of *C. auratus* dorsal muscles in paddy group (PA) and pond group (PO).

	Paddy (PA)	Pond (PO)
C14:0	0.20 ± 0.03	0.21 ± 0.05
C15:0	0.11 ± 0.01	0.07 ± 0.01
C16:0	5.05 ± 0.08 ^b^	3.76 ± 0.19 ^a^
C17:0	0.17 ± 0.01 ^b^	0.10 ± 0.01 ^a^
C18:0	1.46 ± 0.05	1.22 ± 0.05
C23:0	-	0.04 ± 0.01
ΣSFA	6.99 ± 0.05	5.39 ± 0.26
C16:1n7	0.69 ± 0.04 ^b^	0.38 ± 0.04 ^a^
C18:1n9c	3.29 ± 0.34 ^a^	5.68 ± 0.23 ^b^
C20:1	0.27 ± 0.02 ^a^	0.51 ± 0.04 ^b^
C22:1n9	-	0.17 ± 0.01
ΣMUFA	4.25 ± 0.24 ^a^	6.73 ± 0.27 ^b^
C18:2n6c	3.02 ± 0.25 ^a^	5.31 ± 0.24 ^b^
C18:3n6 ##	0.10 ± 0.01	0.11 ± 0.01
C18:3n3 #	0.52 ± 0.07	0.56 ± 0.06
C20:2	0.17 ± 0.01	0.20 ± 0.01
C20:3n6	0.33 ± 0.09	0.41 ± 0.05
C20:3n3	0.08 ± 0.01 ^b^	0.04 ± 0.01 ^a^
C20:4n6 ##	1.98 ± 0.03 ^b^	0.71 ± 0.06 ^a^
C20:5n3 #	0.56 ± 0.03 ^b^	0.25 ± 0.04 ^a^
C22:2n6	0.07 ± 0.01	0.06 ± 0.01
C22:6n3 #	1.52 ± 0.05	1.00 ± 0.09
ΣPUFA	8.35 ± 0.21	8.65 ± 0.06
ΣPUFA (n-3)	2.60 ± 0.12	1.81 ± 0.12
ΣPUFA (n-6)	5.01 ± 0.22	6.02 ± 0.17
ΣPUFA (n-6)/ΣPUFA(n-3)	1.93 ± 0.14	3.35 ± 0.26
Total fatty acids	19.59 ± 0.46	20.77 ± 0.71
*IA*	0.42 ± 0.01 ^b^	0.26 ± 0.01 ^a^
*IT*	0.47 ± 0.01 ^b^	0.34 ± 0.03 ^a^
*PI*	0.41 ± 0.01 ^b^	0.33 ± 0.03 ^a^

- means not detected; # means PUFA (n-3), ## means PUFA (n-6). Values in each row with different letters have significant differences (*p* < 0.05), n = 12.

**Table 6:** Amino acid composition of *C. auratus* dorsal muscles in paddy group (PA) and pond group (PO) (g/100 g muscle).

	Paddy (PA)	Pond (PO)
Asp	1.93 ± 0.09 ^b^	1.51 ± 0.05 ^a^
Thr	0.87 ± 0.04 ^b^	0.53 ± 0.05 ^a^
Ser	0.86 ± 0.07	0.61 ± 0.01
Glu	2.79 ± 0.14	2.36 ± 0.06
Gly	1.16 ± 0.09 ^b^	0.72 ± 0.04 ^a^
Ala	1.23 ± 0.01 ^b^	0.86 ± 0.05 ^a^
Val	0.77 ± 0.07	0.83 ± 0.05
Met	0.47 ± 0.04	0.55 ± 0.07
Ile	0.73 ± 0.07	0.74 ± 0.03
Leu	1.25 ± 0.02 ^b^	0.68 ± 0.10 ^a^
Tyr	0.55 ± 0.05	0.62 ± 0.03
Phe	0.77 ± 0.09	0.81 ± 0.07
Lys	1.90 ± 0.13	1.47 ± 0.01
His	0.58 ± 0.03	0.65 ± 0.09
Arg	1.04 ± 0.09	0.97 ± 0.03
Pro	0.52 ± 0.01	0.52 ± 0.01
∑TAA	17.44 ± 0.82	14.41 ± 0.57
∑EAA	6.77 ± 0.37	5.6 ± 0.31
∑SEAA	1.63 ± 0.11	1.62 ± 0.09
∑NEAA	9.04 ± 0.3 ^b^	7.19 ± 0.21 ^a^
∑DAA	7.11 ± 0.2 ^b^	5.45 ± 0.19 ^a^
∑EAA/∑TAA/%	38.8 ± 0.36	38.83 ± 0.72
∑EAA/∑NEAA/%	74.83 ± 2.31	77.81 ± 3.29
∑DAA/∑TAA/%	40.79 ± 0.57	37.83 ± 0.91

Values in each row with different letters have significant differences (*p* < 0.05), n = 12.

**Table 7:** Amino acid nutritional value evaluation of *C. auratus* dorsal muscles in paddy group (PA) and pond group (PO).

		Paddy (PA)	Pond (PO)
AAS	Thr	118.29 ± 5.18 ^b^	84.96 ± 7.18 ^a^
	Val	83.4 ± 6.83 *	106.55 ± 7.09
	Ile	99.17 ± 7.94	118.32 ± 5.30
	Leu	96.86 ± 4.58 ^b^	62.24 ± 8.35 ^a^ *
	Lys	187.32 ± 10.05	171.28 ± 3.28
	Tyr + Phe	119.4 ± 9.19	152.56 ± 9.79
Fischer ratio		2.09 ± 0.10 ^b^	1.58 ± 0.02 ^a^

* means the first limiting amino acid. Values in each row with different letters have significant differences (*p* < 0.05) n = 12.

## Data Availability

All the data shall be provided upon request.
